# Sensitive Molecules Involved in Spatial Learning and Memory Impairment of Mice Induced by 4.3 GHz Microwave Radiation

**DOI:** 10.3390/biom16070990

**Published:** 2026-07-06

**Authors:** Tingting Qian, Wenjing Cheng, Lequan Song, Ji Dong, Haoyu Wang, Jing Zhang, Li Zhao, Hui Wang, Ruiyun Peng

**Affiliations:** 1 Hengyang Medical College, University of South China, Hengyang 421001, China; 20242126112222@stu.usc.edu.cn; 2 Academy of Military Medical Sciences, Beijing 100850, China; 19156270319@163.com (W.C.); songlq2020@163.com (L.S.); djtjwj@163.com (J.D.); smart106@126.com (H.W.); zhang115614@163.com (J.Z.); lillyliz@163.com (L.Z.)

**Keywords:** microwave radiation, spatial learning and memory, hippocampus, RNA-seq, 4D-DIA proteomics

## Abstract

With the widespread application of microwave technology in communication and medical fields, concerns regarding its biosafety, particularly the effects on the central nervous system, have increased. The brain is considered a sensitive target organ for microwave radiation; however, the molecular mechanisms underlying microwave-induced cognitive impairment remain unclear. The purpose of this study was to evaluate the effects of 4.3 GHz microwave radiation at different power densities on spatial learning and memory in mice, and to identify key molecular changes in the hippocampus associated with cognitive impairment. Mice (male, C57BL/6N) were exposed to 4.3 GHz microwave radiation at power densities of 10 or 30 mW/cm^2^ for 30 min. Spatial learning and memory abilities were assessed using the Morris water maze (MWM). The hippocampal structure was assessed by HE staining at multiple time points following microwave exposure. Integrated RNA-sequencing (RNA-seq) and 4D-data-independent acquisition (4D-DIA) analyses of the hippocampus were performed at 6 h after microwave exposure, and differentially expressed molecules were selected and validated by quantitative polymerase chain reaction (qPCR) and parallel reaction monitoring (PRM). The 4.3 GHz microwave exposure significantly prolonged escape latency in the MWM, indicating impaired spatial learning or navigation ability. Histological examination revealed transient neuronal damage in the hippocampal CA1 and CA3 regions. Multi-omics analysis and subsequent validation revealed molecular alterations. Following microwave radiation, the expression of synaptic plasticity-related genes *Arc* and *Ebf3* was significantly upregulated. At the protein level, significant downregulation was observed for Protein sidekick-2 and IQGAP1, while WNK3 was significantly upregulated. In summary, 4.3 GHz microwave exposure impaired spatial learning or navigation ability, accompanied by structural damage in the hippocampus and molecular alterations in synaptic plasticity-related pathways. *Arc*, *Ebf3*, Protein sidekick-2, WNK3, and IQGAP1 might serve as candidate molecules for understanding and mitigating microwave-induced cognitive deficits.

## 1. Introduction

The widespread application of various electronic instruments and devices in daily life and work has exposed humans to persistent electromagnetic radiation environments, among which the potential health effects of microwave radiation have attracted considerable attention. Modern communication technologies, particularly 5G, rely heavily on the microwave frequency band to achieve higher data transmission rates and reduced latency levels [1]. In the medical field, microwave imaging has demonstrated promising potential to complement or even replace traditional imaging techniques, with particular interest in breast and brain imaging applications [2]. Meanwhile, microwave technology is being actively investigated for therapeutic applications and related bioeffect studies, although its biological safety remains a critical issue [3]. The 4.3 GHz microwaves, which belong to the C-band, are widely utilized owing to their pivotal roles in radar systems, satellite communications, and 5G/6G wireless networks [4]. However, the widespread adoption has also raised growing concerns regarding their biosafety. Accumulating evidence has indicated that microwave radiation at specific power levels could disrupt the nervous, cardiovascular, and other physiological systems. Among them, the brain is considered a sensitive target organ for microwave-induced damage [5,6]. Numerous studies have confirmed that microwave radiation impairs spatial learning and memory in animals and induces structural alterations in brain tissue [7,8]. Specifically, exposure of juvenile mice to 1.85 GHz microwaves results in a significant increase in swimming distance and escape latency in the Morris Water Maze (MWM) test, suggesting impaired spatial learning and memory function [9]. Similarly, mice subjected to 2.1 GHz microwave irradiation exhibit a marked prolongation of path length in the MWM test. These findings indicate that microwave radiation could reduce spatial navigation capabilities and object-location memory in mice [10]. Notably, the impact of microwave radiation on cognitive function varies depending on whether exposure involves single or combined frequencies. A study comparing the effects of S-band (3.5 GHz) and C-band (4.9 GHz) microwaves demonstrated that combined exposure for five weeks (1 h/day, power density 50 W/m^2^) induced cognitive impairment in mice. These behavioral deficits were accompanied by morphological alterations in the hippocampus and amygdala and structural abnormalities in the blood–brain barrier (BBB) and synapses [4]. Another study investigating 1.5 GHz and 4.3 GHz microwaves similarly reported that exposure to either frequency alone impaired spatial learning and memory, with the most severe damage observed following concurrent exposure to both frequencies [11]. These findings suggest that combined exposure to multiple microwave frequency bands might induce more complex biological effects.

Although microwave radiation-induced deficits in spatial learning and memory have been well validated in numerous animal studies, the key molecules related to functional decline remain elusive. Therefore, the present study aimed to evaluate the effects of different doses of 4.3 GHz microwave radiation on learning and memory function in mice and to identify the regulatory targets, thereby providing a theoretical foundation for understanding the biological effects of microwave radiation and for determining potential protective targets.

## 2. Materials and Methods

### 2.1. Animals and Microwave Exposure

A total of 237 male C57BL/6N mice (6–8 weeks old, wild-type) were purchased from Beijing Vital River Laboratory Animal Technology Co., Ltd. (Beijing, China) and housed in a specific pathogen-free (SPF) environment under a 12 h light/dark cycle, with 3–5 mice per cage. Food and distilled water were available ad libitum.

**Figure 1:**
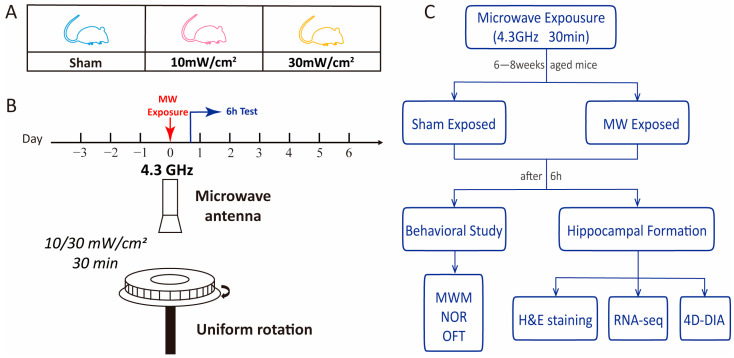
Schematic overview of the experimental design and microwave exposure groups. (**A**) Schematic representation of the mouse model and three experimental groups. (**B**) Timeline of the experimental procedure and microwave setup. (**C**) Flowchart of the experimental workflow.

All mice were allowed to adapt to the experimental environment for at least one week before any procedures. The mice were randomly divided into three groups based on the exposure power density: the sham group, the 10 mW/cm^2^ group, and the 30 mW/cm^2^ group (Figure 1A). Randomisation was performed using a random number table. Mice in the sham group were handled identically but without microwave exposure. All animal procedures were approved by the Academy of Military Medical Sciences on [3 September 2024] (IACUC-DWZX-2024-573). The study was conducted in accordance with the ARRIVE guidelines for the Care and Use of Laboratory Animals.

Inclusion criteria: Healthy appearance and no abnormal behavioral observations after the acclimation period. Exclusion criteria: Obvious health problems or technical failures during data quality checks; these cases will be reported in the results.

For microwave exposure, each mouse was placed separately in a polyethylene container designed to position their heads toward the center, and all measurements and interventions were performed on individual animals independently. These containers were then placed under a C-band microwave radiation source, with the base turntable rotating at a constant speed to ensure uniform irradiation. The configuration of the microwave exposure system and the animal container setup are detailed in Figure 1B. The radiation frequency for all exposure groups was 4.3 GHz (30 min), with average power densities of 10 mW/cm^2^ and 30 mW/cm^2^. The whole-body average Specific Absorption Rate (SAR) values for mice under the two power density conditions were approximately 14.5 W/kg and 37.8 W/kg. With respect to exposure chamber calibration, the source was calibrated both at the beginning and at the end of each experiment, and the power output was continuously monitored during irradiation to guarantee stability. A schematic overview of the entire experimental procedure is presented in Figure 1C.

### 2.2. Behavioral Tests

All behavioral tests were performed between 14:00 and 18:00 daily to avoid time effects. The operation and data analysis of the behavioral experiments were independently performed by researchers blinded to the experimental group allocation.

#### 2.2.1. Morris Water Maze (MWM) Test (*n* = 15 per Group)

During the study design phase, we did conduct an a priori power analysis based on pre-experimental data. Specifically, using the mean difference in escape latency between the sham group and the microwave-exposed group, along with the pooled standard deviation from the pre-experiment, we calculated an effect size of Cohen’s d = 0.64. Setting α = 0.05 and power = 0.80, the required sample size was calculated to be at least 9 animals per group. The sample size we ultimately adopted (*n* = 15 per group) exceeds this calculated value.

The place navigation test was carried out at 6 h to 5 d after microwave radiation. For each trial, mice were individually placed into the water maze from four distinct entry points, facing the pool wall. The average escape latency (AEL), defined as the time required for each mouse to locate the hidden platform within 60 s, was recorded. If the mouse failed to find the platform within 60 s, it was gently guided to the platform, and the escape latency was recorded as 60 s. After each test trial, the mice were dried with a clean towel and put back in their home cages. Once all mice had completed the test from one entry point, the next entry point was utilized sequentially. Following the completion of all four entry point trials per day, the mice were returned to the animal facility. Average escape latencies were analyzed using the Anymaze 6.0 software (Stoelting Co., Wood Dale, IL, USA).

The spatial probe test was conducted 6 d after microwave exposure. The hidden escape platform was removed, and mice from each group were individually placed into the water maze from the northeast (NE) entry point. Their swimming trajectories were recorded continuously for 60 s. Behavioral parameters, including the number of crossings over the original platform location and the time spent in the target quadrant within the 60 s test period, were quantified.

#### 2.2.2. Novel Object Recognition (NOR) Test (*n* = 10 per Group)

During the habituation phase, each mouse was individually put in a white open field (50 × 50 × 40 cm) and allowed to explore freely for 10 min to acclimate to the apparatus and experimental environment. No objects were placed in the arena during this phase. In the learning phase, two identical objects were placed in the open field. The objects were positioned on a diagonal line, with each object 8 cm away from the adjacent walls. Each mouse was allowed to freely explore the two identical objects for 10 min. For the test phase, at 6 h after microwave exposure, one of the two familiar objects was replaced with a novel object that differed distinctly from the familiar one in terms of shape, color, and texture. All other experimental conditions remained consistent with the learning phase. Each mouse was placed in the open field and allowed to explore the familiar and novel objects freely for 5 min. The exploration time for each object was recorded and analyzed using the Anymaze 6.0 software (Stoelting Co., Wood Dale, IL, USA). The discrimination index (DI) for novel object recognition memory was calculated using the formula.

#### 2.2.3. Open Field Test (OFT) (*n* = 10 per Group)

At 6 h after microwave exposure, mice were placed in a white open field (50 × 50 × 40 cm) and allowed to explore freely for 8 min. The arena was virtually divided into a central zone and a peripheral zone for behavioral parameter analysis. Behavioral data, including the number of entries into the central zone and the total time spent in the central and peripheral zones, were recorded and analyzed using the Anymaze 6.0 software (Stoelting Co., Wood Dale, IL, USA). The open-field arena was thoroughly cleaned with 75% ethanol to eliminate residual olfactory cues that might interfere with subsequent trials.

### 2.3. Hematoxylin and Eosin (HE) Staining (n = 5 per Group)

At 1, 3, 7, 14, and 28 d after microwave exposure, five mice per group were randomly selected for tissue sample collection. The left cerebral hemisphere was dissected and immediately immersed in 10% neutral buffered formalin (NBF) for fixation at room temperature. After three weeks of fixation, brain tissues were retrieved and processed. Coronal sections were cut and transferred to labeled embedding cassettes. The tissues were rinsed under running tap water overnight to remove residual formalin, followed by sequential processing in an automatic tissue processor for dehydration, clearing, and paraffin infiltration. After infiltration, the tissues were embedded in paraffin blocks, which were then sectioned into 3 µm thick slices using a microtome. The sections were mounted on poly-L-lysine-coated glass slides to prevent detachment and dried overnight in a 60 °C oven.

The prepared paraffin sections were stained with hematoxylin and eosin. The specific steps were as follows: (1) Deparaffinization and rehydration: Xylene I (10 min) → Xylene II (10 min) → 100% ethanol I (3 min) → 100% ethanol II (3 min) → 95% ethanol (3 min) → 80% ethanol (3 min) → Brief rinse with tap water. (2) Staining: Hematoxylin solution (10 min) → Brief rinse with tap water → Differentiation in 1% hydrochloric acid-ethanol (a few seconds) → Rinse with tap water (20 s) → Bluing in warm tap water (40 s) → 80% ethanol (60 s) → Eosin staining solution (3 min). (3) Dehydration and clearing: 80% ethanol (20 s) → 90% ethanol (20 s) → 95% ethanol I (2 min) → 95% ethanol II (2 min) → 100% ethanol I (3 min) → 100% ethanol II (3 min) → Xylene I (3 min) → Xylene II (3 min) → Xylene III (3 min). (4) Mounting: Sections were mounted with neutral balsam. After natural air-drying, the stained sections were observed under a light microscope (Leica, Wetzlar, Germany), and representative images were captured for histological analysis.

Quantitative analysis of neuronal injury was performed as follows. Five mice were used per group, and three coronal sections per animal were analyzed. The regions of interest, including the dorsal CA1 (dCA1), dorsal CA3 (dCA3), dentate gyrus (DG), ventral CA1 (vCA1), and ventral CA3 (vCA3), were identified according to a mouse brain atlas. For each region, the numbers of morphologically damaged neurons and normal neurons were counted using ImageJ 1.54g software (National Institutes of Health, Bethesda, MD, USA). The percentage of damaged neurons was calculated as (number of damaged neurons/total number of neurons) × 100% in each region. Statistical analysis was then performed on these percentages.

The HE staining procedure and histological image quantification were independently performed by researchers blinded to the experimental group allocation.

### 2.4. RNA-Sequencing (RNA-Seq) (n = 5 per Group)

#### 2.4.1. RNA Extraction and Quality Assessment

Hippocampal tissue samples (*n* = 5 per group) were collected from mice in three groups at 6 h after microwave exposure. Total RNA was extracted and purified using the Trizol method. RNA concentration and purity (OD_260_/OD_280_ ratio) were quantified using a NanoDrop 2000 spectrophotometer (Thermo Fisher Scientific Inc., Waltham, MA, USA). RNA integrity was evaluated using an Agilent 2100 Bioanalyzer (Agilent Technologies Inc., Santa Clara, CA, USA)with the RNA 6000 Nano Kit (Agilent Technologies Inc., Santa Clara, CA, USA). Only RNA samples meeting the quality requirements for library construction were used for subsequent experiments. All samples were processed in the same batch, and no batch effects were detected.

#### 2.4.2. Library Construction and Sequencing

Strand-specific cDNA libraries were constructed using the TruSeq Stranded mRNA LT Sample Prep Kit (Illumina, Inc., San Diego, CA, USA) following the manufacturer’s standard protocol. Briefly, poly (A)-tailed mRNA was enriched from total RNA using oligo (dT)-coated magnetic beads and then randomly fragmented into short segments (≈200 bp) in fragmentation buffer. The fragmented mRNA was reverse-transcribed into first-strand cDNA using random hexamer primers, followed by synthesis of second-strand cDNA with dUTP instead of dTTP to retain strand specificity. After successive steps of end repair (addition of a single A-base), adapter ligation, and PCR amplification (12–15 cycles), strand-specific libraries with an average insert size of approximately 300 bp were generated.

Upon completion of library construction, the fragment size distribution was analyzed and validated using an Agilent 2100 Bioanalyzer (Agilent Technologies Inc., Santa Clara, CA, USA). Library concentration was accurately quantified fluorometrically using the Quant-iT PicoGreen dsDNA Assay Kit (Thermo Fisher Scientific Inc., Waltham, MA, USA), and the effective concentration of each library was further confirmed by quantitative real-time PCR (qPCR) to ensure sequencing quality. Based on the effective concentration and target data output (≥6 Gb per sample), libraries with unique indices were pooled in equal molar amounts, diluted to a final concentration of 2 nM, and denatured to single-stranded DNA. Finally, sequencing was performed on the Illumina NovaSeq 6000 platform (Illumina, Inc., San Diego, CA, USA) with 150 bp paired-end reads (PE150).

#### 2.4.3. Data Filtering and Quality Assessment

After sequencing, raw sequencing data (raw reads) were filtered using Cutadapt (v1.16) and a custom in-house bioinformatics pipeline to generate high-quality clean data. The filtering criteria were defined as follows: (1) Remove low-quality bases (quality score ≤ 30) from both ends of the reads; (2) Remove reads containing adapter contamination; (3) Remove reads with ≥1 ambiguous base (‘N’); (4) Remove reads with a length less than 60 bp. Following quality filtering, high-quality clean reads were obtained. FastQC (v0.10.1) was employed to perform comprehensive quality control (QC) of the clean reads.

#### 2.4.4. Reference Genome Alignment and Transcript Assembly

The clean data were aligned to the mouse reference genome using HISAT2 (v2.1.0) to obtain mapped reads. The alignment results were processed with SAMtools (v1.3.1). Subsequently, StringTie (v1.3.3b) was used to assemble transcripts for each sample. The assembled transcripts from all samples were then merged and compared with the reference genome annotation information to identify known transcripts and discover novel transcripts.

#### 2.4.5. Expression Quantification and Differential Expression Analysis

Gene and transcript expression levels were quantified using StringTie, with the Fragments Per Kilobase of transcript per Million fragments mapped (FPKM) value adopted as the measure of expression abundance. Based on the gene-level count matrix, differential expression analysis between groups was performed using DESeq2 (v1.20.0). Differentially expressed genes (DEGs) were identified using the following stringent criteria: absolute log2 fold change (|log2FC|) > 1 and adjusted *p* < 0.05. Based on these criteria, DEGs were further categorized into up-regulated and down-regulated subsets for subsequent functional analysis.

#### 2.4.6. Functional Annotation and Enrichment Analysis

Functional annotation and enrichment analysis of the identified DEGs were conducted using the Gene Ontology (GO) and Kyoto Encyclopedia of Genes and Genomes (KEGG) databases. The hypergeometric distribution test was employed to calculate the enrichment significance for each functional term, with a *p* < 0.05 set as the threshold for statistically significant enrichment.

GO enrichment analysis covered three core functional categories: biological process (BP), cellular component (CC), and molecular function (MF). KEGG pathway enrichment analysis was performed to annotate DEGs to specific biological pathways and explore potential signaling cascades associated with microwave radiation-induced cognitive impairment.

### 2.5. 4D-Data-Independent Acquisition (4D-DIA) Proteomics (n = 5 per Group)

#### 2.5.1. Protein Extraction and Digestion

Appropriate amounts of tissue samples were lysed in 8 M urea lysis buffer containing protease inhibitors. After thorough lysis, the lysate was centrifuged at 14,100× *g* for 20 min at 4 °C, and the supernatant was collected. Protein concentration was determined using the Bradford assay. Enzymatic digestion was performed using the Qinglian Micro/Universal Proteomics Digestion Kit (Beijing Qinglian Biotech Co., Ltd., Beijing, China). Briefly, an appropriate amount of protein sample was added to an 8-tube strip containing MMB beads and incubated at 37 °C for 30 min. Then, 45 µL of binding buffer was added, followed by incubation at room temperature with shaking for 15 min. After discarding the supernatant, the beads were washed three times. The beads were then resuspended in 40 µL of enzyme working solution and incubated at 37 °C for at least 4 h. Finally, 5 µL of stop solution was added to terminate the reaction, and the samples were lyophilized for subsequent use.

#### 2.5.2. Liquid Chromatography–Mass Spectrometry (LC-MS) Analysis

The lyophilized samples were reconstituted in 10 µL of mobile phase A (0.1% formic acid in water), centrifuged at 14,000× *g* for 20 min at 4 °C, and 400 ng of the supernatant was used for injection. Liquid chromatography was performed using a RIGOL L-3000 system with a separation column (QL-HPLC-100 × 15) at a flow rate of 500 nL/min. Peptides were eluted with a gradient of mobile phase B (80% acetonitrile, 0.1% formic acid) as follows: 0–17 min, 3.5–32% B; 17–18 min, 32–95% B; 18–20 min, 95% B; 20–21 min, 95–1% B; 21–22 min, 1% B. Mass spectrometry analysis was performed on a timsTOF HT (Bruker Corporation, Billerica, MA, USA) equipped with a Captive Spray ion source, operating in Data-Independent Acquisition (DIA) mode. The acquisition parameters were as follows: MS1 scan range, *m*/*z* 300–1500; resolution, 60,000 (*m*/*z* 1222); ion mobility range, 0.70–1.30 V·s/cm^2^; accumulation time, 50 ms; capillary voltage, 1.5 kV; cycle time, 1.23 s.

#### 2.5.3. Data Processing and Database Search

Protein extraction, enzymatic digestion, and mass spectrometry analysis for all samples were performed in a single batch, and no batch effects were detected. Raw mass spectrometry data were searched using Diann v2.2.0 software against the Mus musculus UniProt proteome database (UP000000589, containing 54,742 sequences). The search parameters were as follows: enzyme, Trypsin; maximum missed cleavages, 2; fixed modification, Carbamidomethyl (C); variable modifications, Oxidation (M) and Acetyl (Protein N-term); precursor ion mass tolerance, ±15 ppm; fragment ion mass tolerance, ±0.02 Da. The false discovery rate (FDR) for both peptide and protein identification was controlled at <1%. The raw quantitative values were subjected to median normalization, and technical variability was corrected using the built-in Local Normalization function in Spectronaut. Missing values were imputed using the KNN (k nearest neighbors) imputation method.

#### 2.5.4. Bioinformatics Analysis

Differentially expressed proteins were identified using *t*-tests, with *p* < 0.05 and fold change > 1.2 as the significance thresholds. Functional annotation and enrichment analysis were conducted using the GO, KEGG, Reactome, COG, Pfam, and subcellular localization databases. Enrichment significance was assessed using the hypergeometric distribution test, with significance evaluated by *p*-values or FDR. Protein–protein interaction networks were constructed using the STRING database (combined score ≥ 0.7), and Hub nodes were identified based on their degree values.

### 2.6. Quantitative Real-Time PCR (qPCR) (n = 4 per Group)

Hippocampal tissues were dissected from mice, placed into centrifuge tubes containing 500 µL of Buffer RL, and homogenized. Total RNA was extracted using the FastPure^®^ Cell/Tissue Total RNA Isolation Kit V2 (Vazyme, Beijing, China) according to the manufacturer’s instructions, which included a genomic DNA (gDNA) removal step. The purified RNA was then reverse-transcribed into cDNA using the HiScript^®^ III All-in-one RT SuperMix Perfect for qPCR (Vazyme, Beijing, China). Quantitative real-time PCR (qPCR) was performed to analyze the expression levels of target genes, including *Rasgef1c*, *Atp2b4*, *Cbln1*, *Arc*, and *Ebf3*, using the Taq Pro Universal SYBR qPCR Master Mix (Vazyme, Beijing, China).

Glyceraldehyde-3-phosphate dehydrogenase (GAPDH) was used as the endogenous reference gene for normalizing target gene expression. Control samples were included to exclude potential DNA or RNA contamination. For each gene, at least three replicate reactions were performed per sample. The CT (cycle threshold) values for each gene in each sample were recorded, and melting curve analysis was performed to verify the specificity and reliability of the amplification.

### 2.7. Parallel Reaction Monitoring (PRM) (n = 5 per Group)

Protein digestion was performed using the Qinglian Micro/Universal Proteomics Digestion Kit (Beijing Qinglian Biotech Co., Ltd., MMB-96) according to the manufacturer’s instructions. After digestion, the samples were reconstituted in mobile phase A (0.1% formic acid in water), centrifuged, and 1 µg of the supernatant was injected for analysis. Liquid chromatography was performed using an Easy-nLC 1200 system equipped with a separation column (QL-HPLC-100 × 15). Mobile phase B consisted of 0.1% formic acid in 80% acetonitrile. The peptides were eluted with the following gradient: 0 min (8% B), 5 min (12% B), 35 min (30% B), 44 min (40% B), 45 min (95% B), and 60 min (95% B). Mass spectrometry analysis was performed on an Orbitrap Fusion Lumos mass spectrometer (Thermo Fisher Scientific Inc., Waltham, MA, USA) equipped with a Nanospray Flex™ ion source. The spray voltage was set to 2.2 kV, and the ion transfer tube temperature was 320 °C. The raw mass spectrometry data were analyzed for targeted quantification using Skyline software. The search parameters were set as follows: enzyme, Trypsin; fixed modification, Carbamidomethyl (C); variable modifications, Oxidation (M) and Acetyl (Protein N-terminal); precursor ion mass tolerance, ±15 ppm; fragment ion mass tolerance, ±0.02 Da; maximum missed cleavages; differential expression analysis was performed by comparing the peptide peak areas between the experimental and control groups. Fold change (FC) and *p*-values were calculated, and differentially expressed proteins were identified based on the criteria of *p* < 0.05 and fold change >1.2 or <0.83.

### 2.8. Statistical Analysis

Throughout the entire experiment, no animal death, illness, or technical failure of samples occurred. All allocated animals were included in the final analysis without any exclusion. All data were statistically analyzed using SPSS 26.0 or GraphPad Prism 9.0 software. Comparisons among multiple groups were performed using one-way analysis of variance (ANOVA). For repeated measures data collected at different time points, a two-way repeated measures ANOVA was employed. Post hoc analyses following ANOVA were implemented via LSD, Duncan’s C and two-tailed Dunnett’s *t* tests. Data that met the normality assumption were analyzed using parametric tests, while non-parametric tests were applied when normality was not satisfied. For transcriptome sequencing data, differentially expressed genes were identified using DESeq2 with the criteria of |log_2_FoldChange| > 1 and adjusted *p* < 0.05. For proteomics data, differentially expressed proteins were screened based on the criteria of |Fold Change| ≥ 1.2 and *p* < 0.05. For qPCR results, the relative expression levels of target genes or proteins were first normalized to the internal reference gene (GAPDH) and then calculated as the fold change relative to the sham group. For all statistical tests, *p* < 0.05 was considered statistically significant. Significance levels were indicated as follows: * represents *p* < 0.05, ** represents *p* < 0.01.

## 3. Results

### 3.1. The 4.3 GHz Microwave Exposure Impaired Spatial Learning or Navigation Ability in Mice

**Figure 2:**
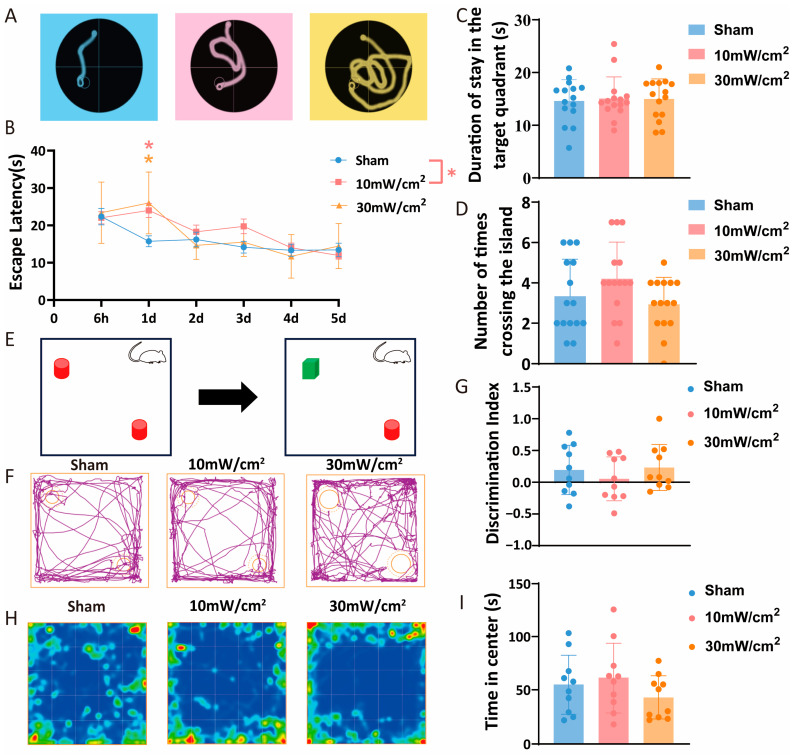
Effects of microwave radiation on cognitive ability in mice. (**A**) Morris water maze test (*n* = 15 mice). Hidden platform tests were conducted at 6 h, 1–5 d after microwave exposure, and representative movement trajectories (**A**) and escape latency (**B**) are shown. Subsequently, the platform was removed, and a probe test was performed. (**C**,**D**) show the time spent in the target quadrant and the number of platform crossings, respectively. (**E**) Schematic diagram of the novel object recognition test (*n* = 10 mice). (**F**) shows the movement trajectories of mice. (**G**) Discrimination index of mice in the novel object recognition test. (**H**) Heat map of the average movement of mice in each group during the open field test (*n* = 10 mice). (**I**) Time spent in the center zone during open field exploration. Compared with the control group, * indicates *p* < 0.05 for the 10 mW/cm^2^ group, and * indicates *p* < 0.05 for the 30 mW/cm^2^ group.

Compared with the sham group, mice in the 10 mW/cm^2^ exposure group displayed significantly prolonged escape latency during the place navigation training (Figure 2B). At 1 d after microwave exposure, escape latency was statistically prolonged in both the 10 mW/cm^2^ and 30 mW/cm^2^ exposure groups relative to the sham group (*p* < 0.05; Figure 2B). No significant differences in swimming speed were observed among the groups, ruling out the potential influence of differences in motor activity (Figure S1). In the probe test, no statistically significant differences were observed among groups in the number of crossings over the original platform location or the percentage of time spent in the target quadrant (all *p* > 0.05; Figure 2C,D), suggesting that spatial reference memory retention was not robustly affected. Collectively, these findings demonstrated that 4.3 GHz microwave exposure induced spatial learning deficits in mice, characterized by impaired consolidation of spatial reference memory during navigation training.

The NOR test was used to assess object recognition memory alterations at 6 h after microwave exposure. Representative exploration trajectories of mice in each group were shown in Figure 2F. Quantitative analysis revealed that there were no statistically significant differences in the novel object discrimination index between the sham group and microwave exposure groups (all *p* > 0.05; Figure 2G). These results suggested that 4.3 GHz microwave exposure did not exert a significant impact on object recognition memory in mice under the tested conditions.

The OFT was conducted 6 h after microwave exposure, and representative locomotor activity heatmaps of mice in the open field arena were shown in Figure 2H. Quantitative analysis revealed that compared with the sham group, there were no statistically significant differences in the percentage of time spent in the central zone among the three groups (all *p* > 0.05; Figure 2I). These findings suggested that 4.3 GHz microwave radiation did not exert a significant impact on anxiety-like behavior in mice.

### 3.2. The 4.3 GHz Microwave Exposure Induced Structural Damage in Mice Hippocampal Subregions

**Figure 3:**
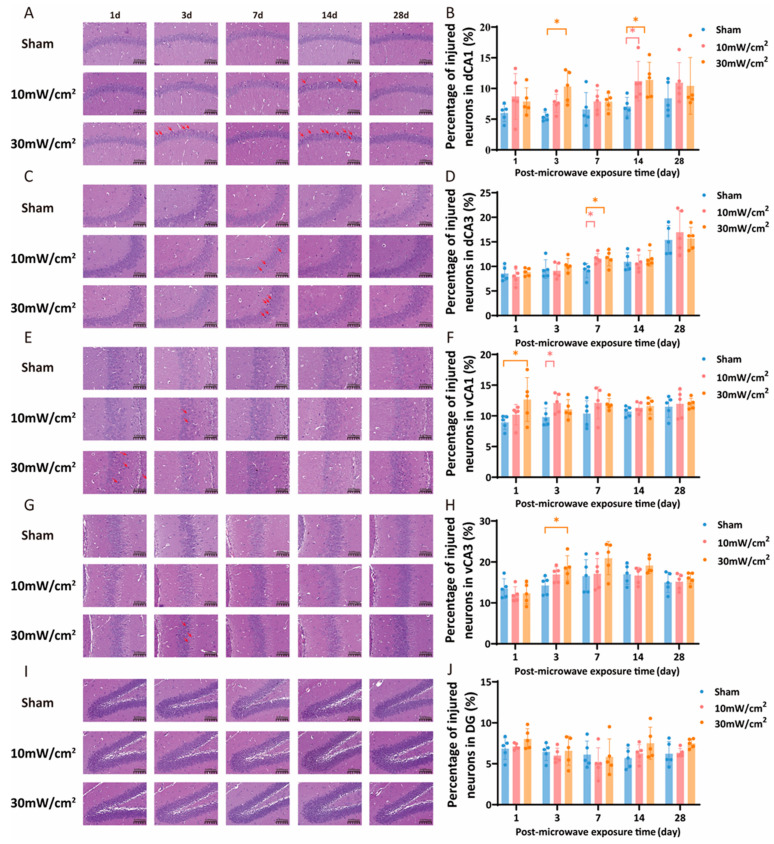
Histomorphological changes in hippocampal subregions after microwave radiation. Representative images showing the dCA1 (**A**), dCA3 (**C**), vCA1 (**E**), vCA3 (**G**), and DG (**I**) region of the hippocampus at different time points after microwave radiation (HE, scale bar = 50 μm). Damaged neurons were indicated by red arrows. The right bar graphs show the quantification of neuronal damage in the dCA1 (**B**), dCA3 (**D**), vCA1 (**F**), vCA3 (**H**), and DG (**J**) region of the hippocampus at different time points after microwave radiation. Compared with the sham group, * indicates *p* < 0.05.

HE staining was performed to evaluate histological changes in hippocampal subregions (dorsal CA1 [dCA1], dorsal CA3 [dCA3], ventral CA1 [vCA1], ventral CA3 [vCA3], and dentate gyrus [DG]) at 1, 3, 7, 14, and 28 d after microwave exposure. In the sham group, the hippocampal subregional structure was morphologically intact, with neurons arranged in an orderly manner, displaying lightly stained nuclei, homogeneous cytoplasm, and distinct nuclear-cytoplasmic boundaries. In contrast, mice in the microwave exposure groups exhibited varying degrees of histological damage, characterized by neuronal pyknosis, hyperchromatic neurons, and enhanced cytoplasmic eosinophilia (Figure 3).

Structures of the dCA1 region at different time points after microwave exposure were shown in Figure 3A. At 3, 7, and 14 d after microwave exposure, the percentage of damaged neurons increased in all exposure groups, with the most severe damage observed at 14 d. Changes in dCA3 region at different time points after microwave exposure were shown in Figure 3C. At 7 d, compared with the sham group, the number of damaged neurons increased in both the 10 and 30 mW/cm^2^ groups. The vCA1 region at different time points after microwave exposure was shown in Figure 3E. Damage was observed as early as 1 d post-exposure, manifested as an increase in damaged neurons in the 30 mW/cm^2^ group. HE staining results of the vCA3 region at different time points after microwave exposure were shown in Figure 3G. At 3 d after microwave exposure, an increase in damaged neurons was observed in the 30 mW/cm^2^ group. Overall, all exposure groups exhibited varying degrees of structural damage in hippocampal subregions (dCA1, dCA3, vCA1, vCA3). Statistical analysis revealed a significant increase in the number of pyknotic and hyperchromatic neurons (*p* < 0.05), as shown in Figure 3. In most brain regions, damage began to appear at 3 d, was most pronounced at 3 and 7 d, followed by a trend of recovery, with complete restoration observed at 28 d. We also examined neuronal changes in the dentate gyrus (DG) region and found no significant damage in the hippocampal DG (Figure 3I,J).

### 3.3. The 4.3 GHz Microwave Exposure Altered mRNA Expression in the Mouse Hippocampus

Quality inspection was performed on mouse hippocampal tissues. The quality control results for each sample after processing are shown in Table S1. Q20 > 90% and Q30 > 85% indicated good RNA sequencing quality and high reliability, making the data suitable for subsequent analyses. The inter-sample Pearson correlation heatmap (Figure S3) showed that samples within the same group exhibited high correlation, indicating good data reproducibility and no outlier samples. The raw sequencing data are not currently publicly available, but can be obtained from the corresponding author upon reasonable request.

**Figure 4:**
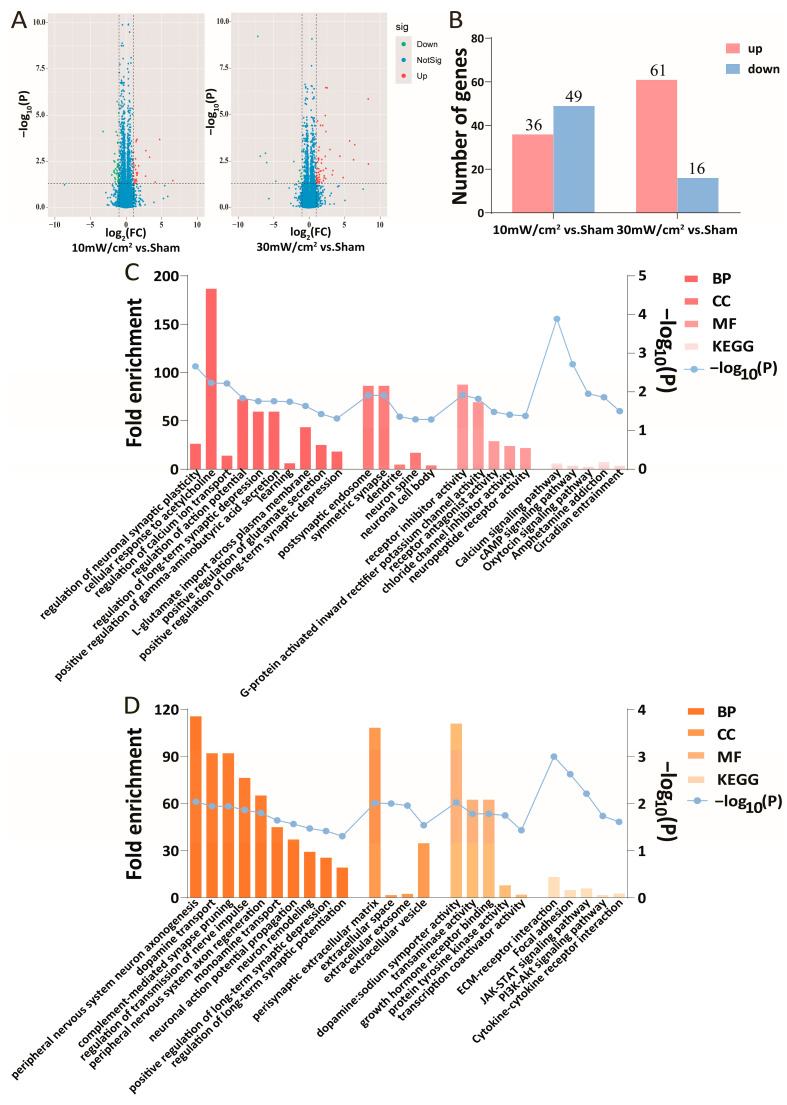
Transcriptomic analysis of hippocampal tissue after microwave irradiation. (**A**) Volcano plot of differential mRNAs in the HPC brain region after microwave radiation at different power densities. (Upregulated genes in red, downregulated genes in green.) (**B**) Quantitative statistics of differential mRNAs. (**C**) Enrichment analysis (GO and KEGG) of differentially expressed mRNAs in hippocampal tissue between the sham group and the 10 mW/cm^2^ group. (**D**) Enrichment analysis (GO and KEGG) of differentially expressed mRNAs in hippocampal tissue between the sham group and the 30 mW/cm^2^ group.

A substantial number of genes with statistically significant expression alterations were found, indicating distinct transcriptional profiles induced by 4.3 GHz microwave radiation. Compared with the sham group, the 10 mW/cm^2^ group had 36 statistically significant up-regulated differentially expressed genes (DEGs) and 49 down-regulated DEGs. The 30 mW/cm^2^ group exhibited 61 significantly up-regulated DEGs and 16 significantly down-regulated DEGs (Figure 4B).

In the 10 mW/cm^2^ group, the enriched BP primarily included regulation of neuronal synaptic plasticity, dorsal spinal cord interneuron posterior axon guidance, regulation of cell communication by electrical coupling, protein export from the nucleus, lateral motor column neuron migration, and so on. Enriched CC included postsynaptic endosome, symmetric synapse, dendrite, neuron spine, and neuronal cell body. Enriched MF included nitric-oxide synthase binding, voltage-gated potassium channel activity involved in atrial cardiac muscle cell action potential repolarization, G-protein activated inward rectifier potassium channel activity, calmodulin binding, and neuropeptide receptor activity. KEGG pathway enrichment analysis based on the differentially expressed mRNAs identified several significant pathways, including the calcium signaling pathway, cAMP signaling pathway, oxytocin signaling pathway, amphetamine addiction, and circadian entrainment (Figure 4C).

In the 30 mW/cm^2^ group, the enriched BP primarily included dorsal spinal cord interneuron posterior axon guidance, peripheral nervous system neuron axonogenesis, dopamine transport, complement-mediated synaptic pruning, regulation of transmission of nerve impulse, and so on. Enriched CC included the presynaptic extracellular matrix, extracellular space, extracellular exosomes, and extracellular vesicles. Enriched MF included dopamine: sodium symporter activity, transaminase activity, growth hormone receptor binding, protein tyrosine kinase activity, and transcriptional coactivator activity. KEGG pathway enrichment analysis identified several significant pathways, including ECM-receptor interaction, focal adhesion, the JAK-STAT signaling pathway, PI3K-Akt signaling pathway, and cytokine–cytokine receptor interaction (Figure 4D).

**Figure 5:**
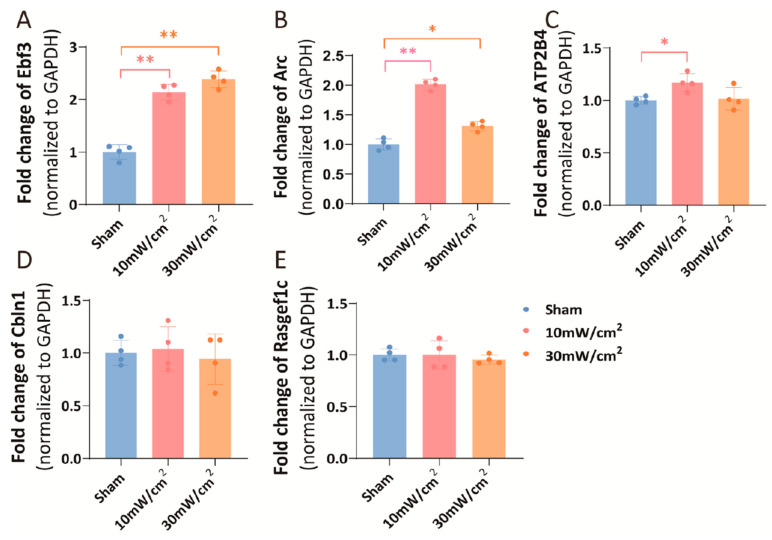
qPCR analysis of differential gene expression in hippocampal tissue after microwave radiation at different power densities. (**A**) Fold change in *Ebf3* in the hippocampus. (**B**) Fold change in *Arc* in the hippocampus. (**C**) Fold change in *Atp2b4* in the hippocampus. (**D**) Fold change in *Rasgef1c* in the hippocampus. (**E**) Fold change in *Cbln1* in the hippocampus. Compared with the sham group, * indicates *p* < 0.05, ** indicates *p* < 0.01.

Five DEGs (*Rasgef1c*, *Atp2b4*, *Cbln1*, *Arc*, and *Ebf3*) directly associated with spatial learning and memory processes were selected for qPCR verification at 6 h after microwave exposure. Candidate genes were chosen based on their fold change (FC) values and their relevance to neuronal plasticity or memory formation. According to qPCR results, *Ebf3* and *Arc* exhibited statistically significant up-regulation in all microwave radiation groups (10 mW/cm^2^ and 30 mW/cm^2^) compared with the sham group (Figure 5A,B). In the 10 mW/cm^2^ radiation group, *Atp2b4* also showed significant up-regulation (Figure 5C). No statistically significant differences in *Cbln1* or *Rasgef1c* expression levels were detected between the radiation groups and the sham group (Figure 5D,E).

### 3.4. The 4.3 GHz Microwave Exposure Altered Protein Expression in Mouse Hippocampus

The global clustering heatmap (Figure S3A) showed that samples within the same group were highly clustered, with clear inter-group differences, demonstrating good data reproducibility.

**Figure 6:**
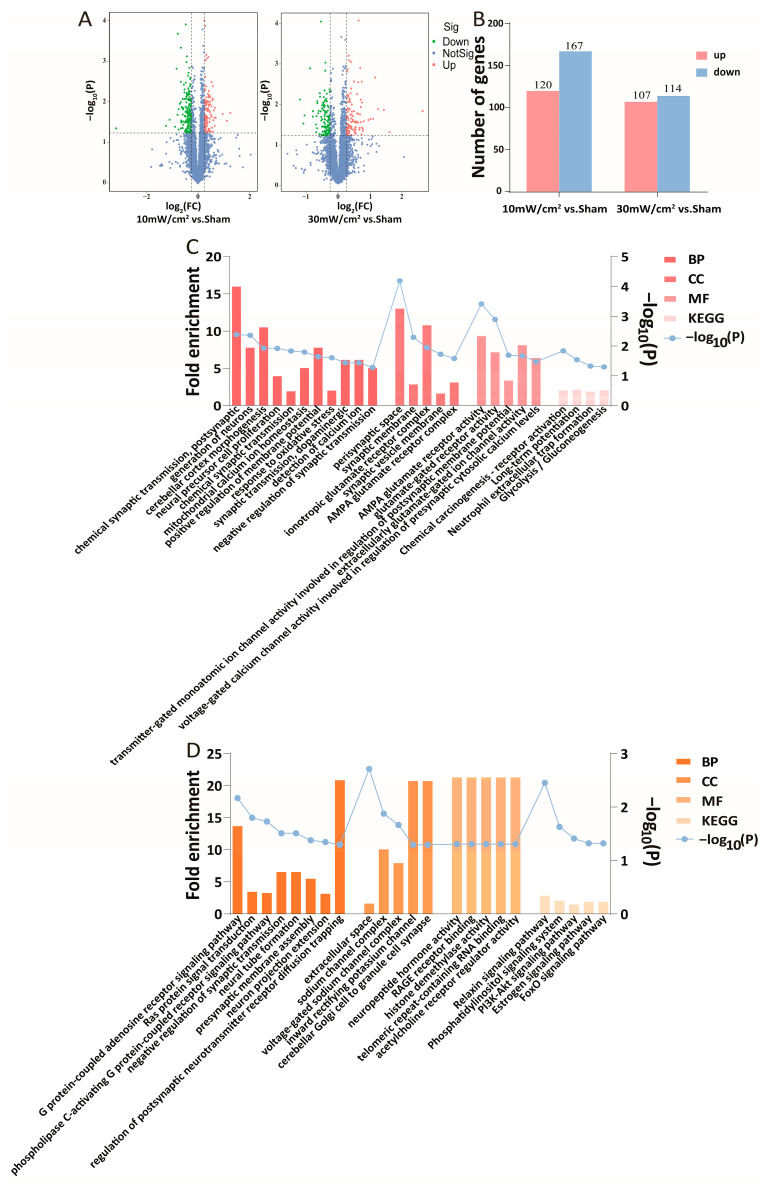

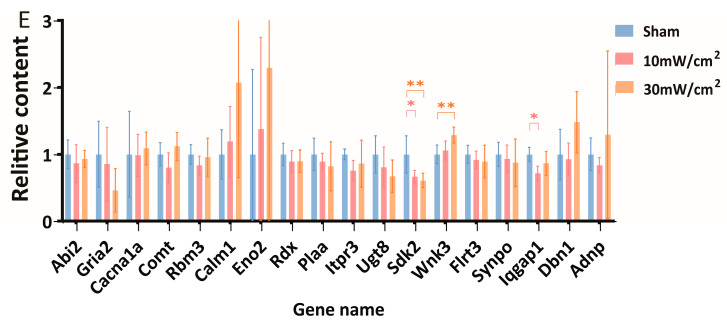
Proteomic analysis of hippocampal tissue after microwave irradiation. (**A**) Volcano plot of differential proteins in the HPC brain region after microwave radiation at different power densities. (Upregulated proteins in red, downregulated proteins in green).) (**B**) Quantitative statistics of differential proteins identified by quantitative proteomics in the HPC brain region. (**C**) Enrichment analysis (GO and KEGG) of differentially expressed proteins in hippocampal tissue between the sham group and the 10 mW/cm^2^ group. (**D**) Enrichment analysis (GO and KEGG) of differentially expressed proteins in hippocampal tissue between the sham group and the 30 mW/cm^2^ group. (**E**) Protein validation by PRM. Compared with the sham group, * indicates *p* < 0.05, ** indicates *p* < 0.01.

A total of 508 statistically significant DEPs were identified across the two exposure doses, including 227 up-regulated proteins and 281 down-regulated proteins. Compared with the sham group, the 10 mW/cm^2^ group had 120 up-regulated and 167 down-regulated proteins; the 30 mW/cm^2^ group had 107 up-regulated and 114 down-regulated proteins (Figure 6B).

GO and KEGG enrichment analyses were performed on the differentially expressed proteins between the 10 mW/cm^2^ group and the sham group to identify functional terms and pathways. The most significantly enriched BP terms were closely related to chemical synaptic transmission, postsynaptic, generation of neurons, cerebellar cortex morphogenesis, neural precursor cell proliferation, and so on. Enriched CC terms were predominantly localized to presynaptic space, synaptic membrane, ionotropic glutamate receptor complex, synaptic vesicle membrane, and AMPA glutamate receptor complex. Key enriched MF terms included AMPA glutamate receptor activity, glutamate-gated receptor activity, transmitter-gated monoatomic ion channel activity involved in the regulation of postsynaptic membrane potential, extracellular glutamate-gated ion channel activity, and so on. KEGG pathway analysis identified several statistically significant pathways associated with neuronal function and memory formation, including chemical carcinogenesis-receptor activation, long-term potentiation, neutrophil extracellular trap formation, and glycolysis/gluconeogenesis (Figure 6C).

Compared with the sham group, the enriched BP term in the 30 mW/cm^2^ group included the G protein-coupled adenosine receptor signaling pathway, Ras protein signal transduction, phospholipase C-activating G protein-coupled receptor signaling pathway, negative regulation of synaptic transmission, neural tube formation, and so on. Enriched CC included the extracellular space, sodium channel complex, voltage-gated sodium channel complex, inward rectifier potassium channel, and cerebellar Golgi cell to granule cell synapse. Enriched MF included neuropeptide hormone activity, RAGE receptor binding, histone demethylase activity, telomeric repeat-containing RNA binding, and acetylcholine receptor regulator activity. KEGG pathway analysis of the differentially expressed proteins identified several pathways, including the Relaxin signaling pathway, phosphatidylinositol signaling system, PI3K-Akt signaling pathway, estrogen signaling pathway, and FoxO signaling pathway (Figure 6D).

To validate the reliability of 4D-DIA proteomics results and prioritize key functional proteins implicated in spatial learning and memory impairment, PRM quantitative proteomic analysis was performed on DEPs from the microwave radiation group and the sham group at 6 h post-exposure. PRM targets were selected based on their functional relevance to neuronal function and differential expression magnitude (|FC| ≥ 1.2, *p* < 0.05). Integration of 4D-DIA and PRM proteomics data revealed that the expression levels of sidekick-2 and IQGAP1 were significantly down-regulated in the mouse hippocampal region of the radiation group, compared with the sham group (Figure 6E).

## 4. Discussion

With the rapid advancement and widespread adoption of microwave technology, human exposure to microwave electromagnetic fields in daily life has increased markedly. Consequently, public concerns over the safety of non-ionizing radiation have heightened, leading to increased scrutiny of the potential health risks posed by microwave radiation. As the brain is one of the most sensitive target organs for microwave radiation, systematically investigating its impact on the nervous system is therefore of paramount significance. Extensive animal studies have demonstrated that exposure to microwaves of diverse frequencies and intensities induces neurocognitive impairments in rodents, including spatial learning and memory deficits, attention impairments, anxiety-like behaviors, and hippocampal structural damage [12,13,14]. These behavioral alterations are frequently accompanied by hippocampal neuronal structural damage, decreased cortical excitability, impaired synaptic plasticity, and neuronal ultrastructural abnormalities, indicating that microwave radiation exerts direct or indirect toxic effects on the central nervous system (CNS) [15]. Mechanistic investigations have further revealed that these neurotoxic effects may be linked to multiple pathological processes, including compromised synaptic plasticity, inhibition of the Brain-Derived Neurotrophic Factor (BDNF)/Tropomyosin receptor kinase B (TrkB) signaling pathway, dysregulated N-methyl-D-aspartate Receptor (NMDAR) expression, neuroinflammation driven by microglial activation, and perturbations in epigenetic regulation [5,6]. Moreover, the specific molecular and cellular mechanisms underlying microwave radiation-induced neurocognitive dysfunction remain incompletely understood. A comprehensive understanding of these underlying mechanisms is essential for evaluating the potential risks of radiofrequency electromagnetic field (RF-EMF) exposure to human cognitive health and for developing targeted protective strategies.

Spatial learning and memory are core components of high cognitive function, critically reliant on the structural and functional integrity of neural circuits as well as synaptic plasticity in brain regions such as the hippocampus and prefrontal cortex [16]. Microwave radiation is known to interfere with the normal function of these complex neural processes via both thermal and non-thermal mechanisms, ultimately leading to cognitive impairments [5,17]. The MWM results of our study demonstrated that exposure to 4.3 GHz microwaves at 10 mW/cm^2^ and 30 mW/cm^2^ significantly prolonged escape latency in mice compared with the sham group, indicating impaired spatial learning ability, which was consistent with previous reports of spatial learning deficits following microwave exposure in other frequency bands [13,18,19].

The underlying mechanism for this discrepancy may involve the specific exposure dose, unique characteristics of the 4.3 GHz frequency, or region-specific sensitivity of distinct hippocampal functional subregions to microwave radiation [20,21]. Furthermore, we also detected changes in the novel object recognition and open field tests of mice and found no significant differences. Overall, the behavior experiments indicated specific impairments in spatial reference memory.

Learning and memory function is associated with hippocampal structure. As a central hub for spatial memory and the integration/processing of new memory information [22], the hippocampus possesses distinct laminar architecture and plays important roles in memory encoding, consolidation, and retrieval.

HE staining results revealed that after microwave exposure, significant neuronal damage occurred in both the dorsal and ventral CA1 and CA3 regions of the hippocampus. Notably, no significant damage was observed in the DG region, which plays a critical role in pattern separation and encoding new information, whereas the CA3 and CA1 regions are more involved in memory encoding and retrieval [22]. Furthermore, the 6–8-week-old experimental animals are characterized by active neurogenesis in the dentate gyrus [23]. Microwave-induced cellular stress in the DG may have been rapidly compensated by an influx of newborn neurons, thereby masking persistent histological injury. This might explain the lack of significant differences in the spatial probe test. Based on this, our study further revealed differential sensitivity of various hippocampal subregions to microwave-induced damage within the same frequency band. The results showed that the CA1 region, particularly the vCA1, exhibited damage as early as 1-day post-exposure, suggesting that this area might be more sensitive to microwave radiation and that its injury could be a key factor contributing to the early decline in learning ability.

To investigate the molecular mechanisms underlying cognitive impairment induced by microwave radiation, we conducted integrated transcriptomic and proteomic profiling of mouse hippocampal tissue.

The results showed that microwave exposure disrupted multiple biological processes closely associated with learning and memory, such as the calcium signaling pathway, the cAMP signaling pathway, long-term potentiation, and synaptic-related functions.

In the present study, KEGG pathway analysis revealed that microwave exposure was associated with alterations in several signaling pathways, including the PI3K-Akt pathway and pathways related to synaptic plasticity. However, it is important to note that these findings were derived from an exploratory discovery-phase analysis. We did not perform functional intervention experiments to establish a causal relationship between the activation or inhibition of these pathways and the observed spatial learning impairment. Future work using gain-of-function or loss-of-function approaches will be necessary to determine the direct roles of these pathways in microwave-induced cognitive deficits.

Through validation experiments on the transcriptomic and proteomic data, we identified several key molecules. The mRNA expression of *Arc* and *Ebf3* and the protein expression of WNK3 were significantly up-regulated after radiation, while the expression of protein sidekick-2 and IQGAP1 was significantly decreased after radiation.

Although RNA-seq identified multiple differentially expressed genes, subsequent qPCR validation confirmed concordant directional changes only for *Arc* and *Ebf3*. The expression direction of *Atp2b4* was opposite to that predicted by RNA-seq, while *Rasgef1c* and *Cbln1* did not reach statistical significance in qPCR. Several factors may account for these discrepancies: RNA-seq is a high-throughput screening method that has higher noise for low-abundance transcripts, whereas qPCR offers higher sensitivity and a broader dynamic range; differences in RNA extraction, reverse transcription efficiency, and cellular composition between the two platforms may also contribute to inconsistencies. Therefore, in the present study, we only consider *Arc* and *Ebf3* as reliably cross-validated candidate genes. The other candidate genes should be interpreted with caution and require further validation in independent samples.

*Arc*, an immediate early gene, is tightly regulated by neuronal activity and plays a central role in synaptic plasticity, long-term potentiation, and memory consolidation [24]. The up-regulation of *Arc* may represent a compensatory response of neurons to microwave-induced injury, attempting to maintain or repair impaired synaptic function. *Ebf3* regulates neuronal differentiation, migration, and cholinergic neuron development, and is associated with neurogenesis and differentiation [25]. There is now conclusive evidence that heterozygous loss-of-function variants of *Ebf3* are clearly associated with autism spectrum disorder and neurodevelopmental disorders, suggesting that *Ebf3* plays a critical role in neurodevelopment [26]. The up-regulation may reflect the initiation of repair processes following injury. Protein sidekick-2, as an adhesion molecule that promotes retinal layer-specific synaptic connectivity and is specifically involved in forming neural circuits for motion detection, plays a critical role in nervous system development and synaptic connectivity [27,28]. Dysfunction of sidekick-2 may lead to disruption of retinal layer-specific synaptic connectivity and impaired formation of neural circuits for motion detection, ultimately affecting synaptic plasticity and neural network function. WNK3 regulates GABAergic signaling by influencing intracellular chloride homeostasis, thereby playing a role in neuronal morphological development and excitability [29]. However, dysfunction of WNK3 disrupts chloride homeostasis by dysregulating KCC2 phosphorylation, a site critically regulated during the development of synaptic inhibition [30]. IQ-domain GTPase activating protein 1 (IQGAP1), as a scaffolding protein, may play a complex regulatory role in neurobiological processes such as neuronal morphogenesis, axon guidance, and synaptic plasticity. Moreover, studies have shown that the IQGAP1/extracellular regulated protein kinases (ERK) signaling pathway may influence memory formation by regulating histone deacetylase2 (HDAC2)-mediated histone modification [31,32].

In addition, accumulating evidence links oxidative stress and neuroinflammation to microwave-induced cognitive impairment [33]. Studies have shown that compounds with antioxidant properties effectively improve learning and memory abilities in radiation-exposed rats by alleviating oxidative stress [34]. Microwave exposure elevates mitochondrial ROS and suppresses KEAP1-Nrf2, with PINK1-Parkin restoration rescuing these effects [35]. Although direct oxidative markers are rarely measured, NLRP3 inflammasome-driven pyroptosis [4], NF-κB upregulation, and reduced BDNF-TrkB-CREB signaling [36] suggest upstream oxidative injury. In the hippocampus, microwave activates microglia and raises pro-inflammatory cytokines while reducing the anti-inflammatory cytokine IL-4 [36,37]; it also triggers NLRP3-mediated caspase-1 cleavage in CA3/amygdala with BBB disruption [36]. Another study reported microwave downregulates m^6^A, reducing TrkB mRNA stability and BDNF, thus inhibiting neural stem cell plasticity [6]. The NLRP3 inflammasome is a key hub linking oxidative stress and neuroinflammation [38]. Therefore, oxidative stress and neuroinflammation likely act upstream of, or synergistically with, the observed changes in *Arc*, *Ebf3*, IQGAP1, and sidekick-2, contributing to hippocampal CA1/CA3 structural damage and spatial learning deficits. Integrating these mechanistic links provides a more comprehensive understanding of microwave-induced neurotoxicity.

In summary, this study evaluated the effects of 4.3 GHz microwave exposure at different power densities (10 and 30 mW/cm^2^) on spatial learning and memory abilities in mice, comprehensively delineating the neurobehavioral, structural, and molecular neurological impacts of 4.3 GHz microwave radiation. Our findings demonstrated that 4.3 GHz microwave exposure impaired spatial learning or navigation ability in mice and induced structural damage in the hippocampal CA1 and CA3 regions, but had no significant effects on object recognition memory or anxiety-like behavior. Through transcriptomic and proteomic analyses, we identified a series of DEGs and DEPs associated with synaptic plasticity, calcium signaling, and neurodevelopment. Among these, the up-regulation of *Arc*, *Ebf3*, and WNK3, coupled with the down-regulation of sidekick-2 and IQGAP1, may contribute to microwave-induced cognitive impairment.

The head-directed microwave exposure model approximates certain near-field human exposure scenarios but does not fully replicate real-world conditions involving variable power, distance, duration, and movement. Furthermore, intervention experiments are still needed to establish a direct causal link between candidate molecules and microwave-induced cognitive deficits. In the future, we need to explore the relationships among differentially expressed proteins within hippocampal subregions. Nevertheless, the key molecular pathways identified are highly conserved across mammals, suggesting potential relevance to human neurobiology. Further studies in diverse models and, where possible, human epidemiological or experimental setups are needed to assess translational significance.

## 5. Conclusions

In conclusion, this study demonstrated that 4.3 GHz microwave exposure could impair spatial learning and memory in mice. The underlying mechanism might involve the induction of neuronal damage in the hippocampal CA1 and CA3 regions, along with the impairment of synaptic plasticity and dysregulation of calcium homeostasis, which were associated with changes in *Arc*, *Ebf3*, Protein sidekick-2, IQGAP1, and WNK3. These findings provided novel insights into the neurobiological effects of microwave radiation and laid a critical theoretical foundation for assessing the biosafety of 4.3 GHz microwave exposure and identifying potential therapeutic targets for mitigating its adverse neurological impacts.

## Data Availability

The data that support the findings of this study are available from the corresponding author upon reasonable request.

## Supplementary Materials

The following supporting information can be downloaded at https://www.mdpi.com/article/10.3390/biom16070990/s1: Figure S1: Effects of microwave radiation on kinematic parameters of mice in the Morris water maze. Dynamic alterations in the average swimming distance (A) and swimming speed (B) of mice after exposure at different power densities; Figure S2: Representative high-magnification images of HE staining in the mouse hippocampus. Representative images showing the dCA1 (A), dCA3 (B), vCA1 (C) and vCA3 (D) region of the hippocampus at different time points after microwave radiation (HE, scale bar = 20 μm). Damaged neurons were indicated by red arrows; Figure S3: Cluster heatmap of differentially expressed proteins (A) and genes (B) in the hippocampus following microwave radiation at distinct power densities. Darker red color corresponds to higher protein expression level, while darker blue color corresponds to lower protein expression level; Table S1: Quality control (QC) results for the processed hippocampal tissue data.
