# Supplementary material for: Sensitive Molecules Involved in Spatial Learning and Memory Impairment of Mice Induced by 4.3 GHz Microwave Radiation
Source: Biomolecules. 2026 Jul 6;16(7):990. doi: 10.3390/biom16070990 (PMC13406997; doi:10.3390/biom16070990)
Supplement: Supplementary file 1 [file biomolecules-16-00990-s001.zip › biomolecules-4323479-supplementary.pdf]

Supplementary Materials for

Sensitive Molecules Involved in Spatial Learning and Memory Impairment of Mice Induced by 4.3 GHz Microwave Radiation

Tingting Qian , Wenjing Cheng , Lequan Song , Ji Dong , Haoyu Wang , Jing Zhang ,

Li Zhao , Hui Wang and Ruiyun Peng

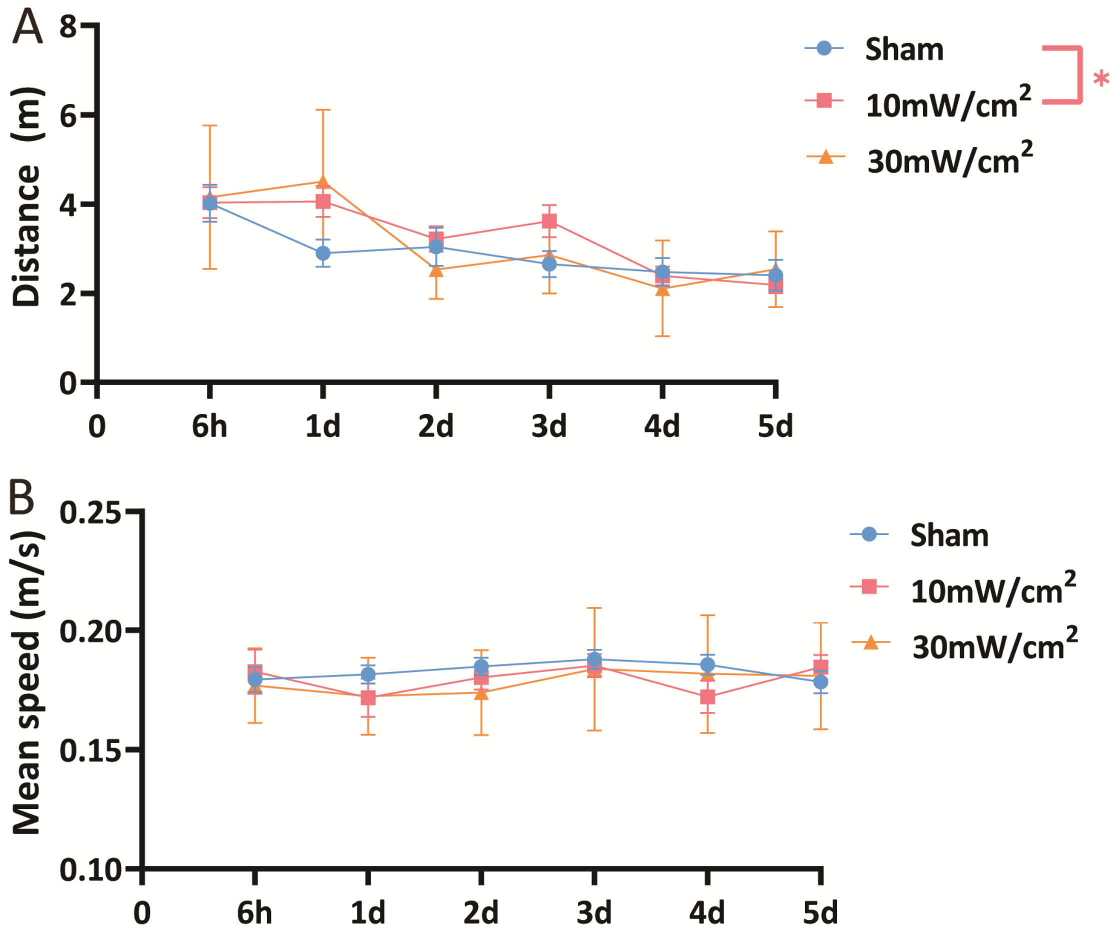

Figure S1. **Effects of microwave radiation on kinematic parameters of mice in the Morris water maze.**

Dynamic alterations in the average swimming distance (A) and swimming speed (B) of mice after exposure at different power densities.

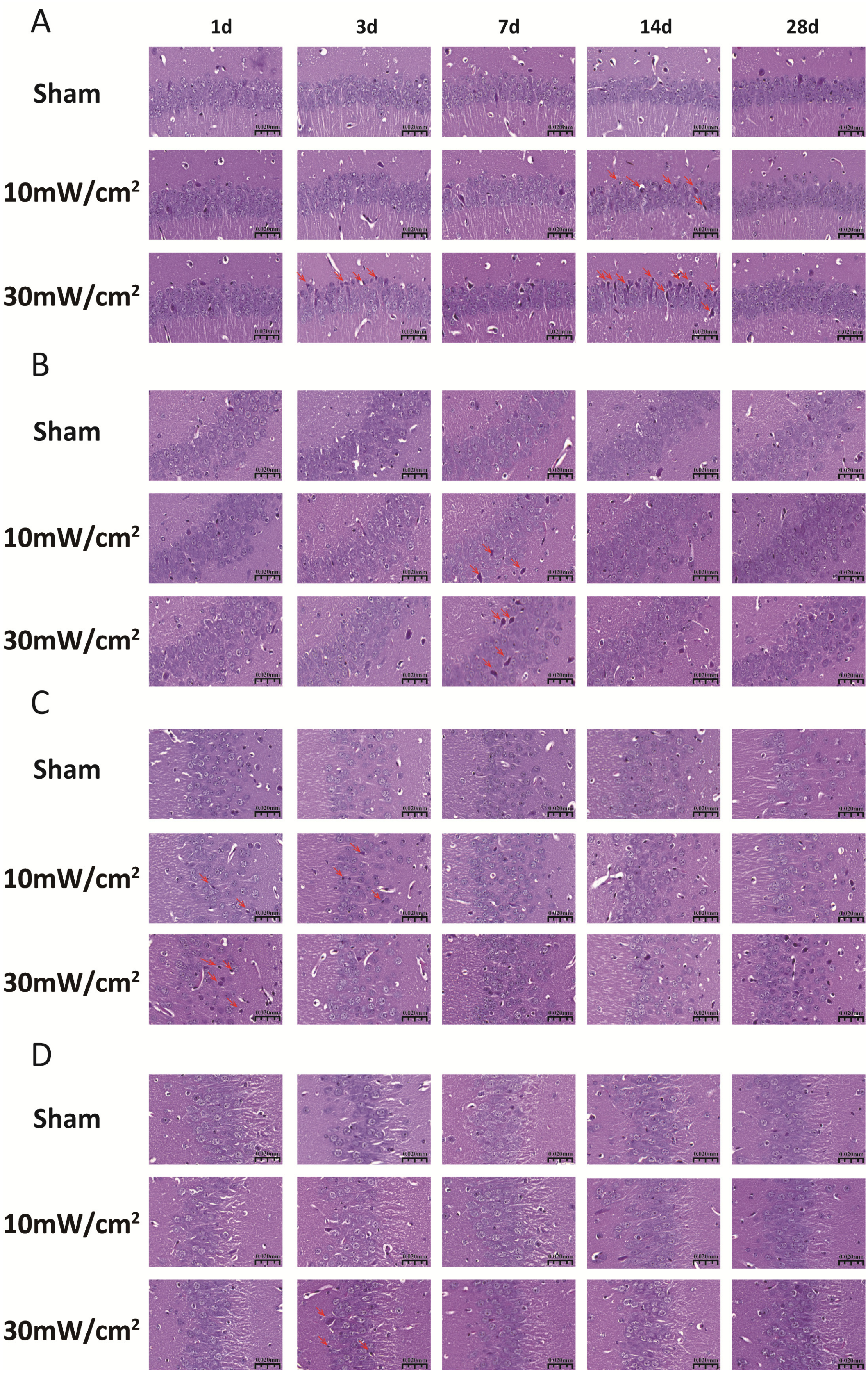

Figure S2. **Representative high-magnification images of HE staining in the mouse hippocampus.**

Representative images showing the dCA1 (A), dCA3 (B), vCA1 (C) and vCA3 (D) region of the hippocampus at different time points after microwave radiation (HE, scale bar = 20 μm). Damaged neurons were indicated by red arrows.

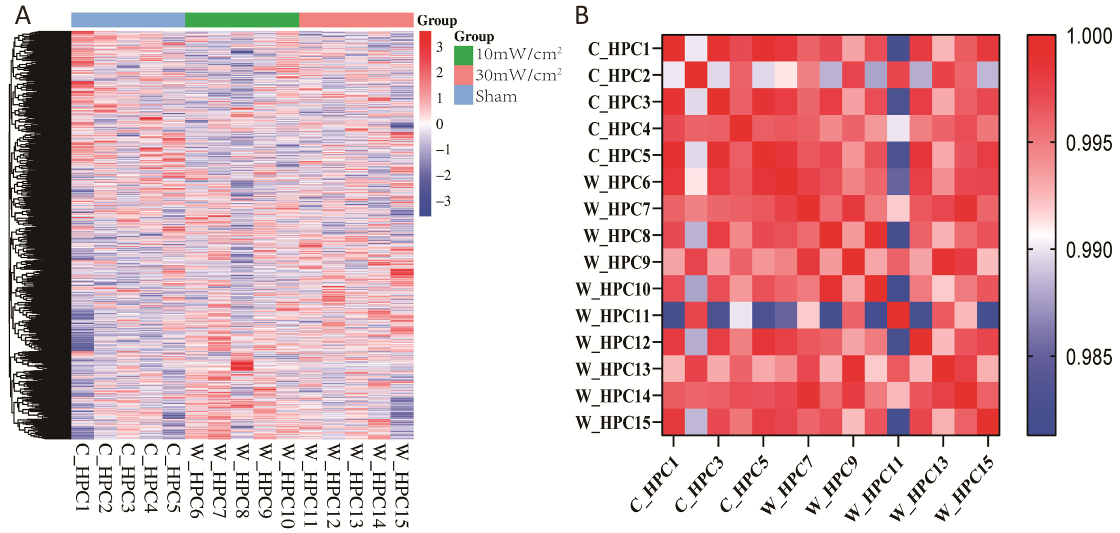

Figure S3. Cluster heatmap of differentially expressed proteins (A) and genes (B) in the hippocampus following microwave radiation at distinct power densities.

Darker red color corresponds to higher protein expression level, while darker blue color corresponds to lower protein expression level.

Table S1. Quality control (QC) results for the processed hippocampal tissue data.

Sample	Raw Reads (M)	Raw Bases (G)	Raw Q20(G)	Raw Q30(G)	Clean Reads(M)	Clean Bases(G)	Clean Q20(G)	Clean Q30(G)	Clean GC
C_HPC1	50.650	7.567	7.511(99.3%)	7.284(96.3%)	50.607(99.9%)	7.551(99.8%)	7.498(99.3%)	7.274(96.3%)	49.55%
C_HPC2	36.874	5.508	5.469(99.3%)	5.311(96.4%)	36.843(99.9%)	5.497(99.8%)	5.460(99.3%)	5.303(96.5%)	49.68%
C_HPC3	37.525	5.603	5.563(99.3%)	5.398(96.3%)	37.492(99.9%)	5.591(99.8%)	5.553(99.3%)	5.390(96.4%)	49.64%
C_HPC4	38.888	5.809	5.767(99.3%)	5.595(96.3%)	38.854(99.9%)	5.797(99.8%)	5.757(99.3%)	5.587(96.4%)	49.73%
C_HPC5	40.757	6.087	6.044(99.3%)	5.869(96.4%)	40.721(99.9%)	6.074(99.8%)	6.033(99.3%)	5.861(96.5%)	49.68%
W_HPC6	39.346	5.874	5.832(99.3%)	5.661(96.4%)	39.313(99.9%)	5.862(99.8%)	5.822(99.3%)	5.653(96.4%)	49.56%
W_HPC7	36.372	5.431	5.393(99.3%)	5.236(96.4%)	36.341(99.9%)	5.420(99.8%)	5.383(99.3%)	5.228(96.5%)	49.66%
W_HPC8	42.209	6.306	6.262(99.3%)	6.075(96.3%)	42.174(99.9%)	6.294(99.8%)	6.251(99.3%)	6.067(96.4%)	49.79%
W_HPC9	38.072	5.685	5.645(99.3%)	5.482(96.4%)	38.039(99.9%)	5.673(99.8%)	5.635(99.3%)	5.474(96.5%)	49.85%
W_HPC10	33.800	5.049	5.012(99.3%)	4.864(96.3%)	33.775(99.9%)	5.039(99.8%)	5.004(99.3%)	4.858(96.4%)	49.77%
W_HPC11	36.186	5.403	5.365(99.3%)	5.209(96.4%)	36.154(99.9%)	5.391(99.8%)	5.355(99.3%)	5.201(96.5%)	49.79%
W_HPC12	50.723	7.577	7.524(99.3%)	7.306(96.4%)	50.678(99.9%)	7.561(99.8%)	7.510(99.3%)	7.295(96.5%)	49.47%
W_HPC13	43.364	6.478	6.432(99.3%)	6.246(96.4%)	43.327(99.9%)	6.464(99.8%)	6.421(99.3%)	6.237(96.5%)	49.90%
W_HPC14	39.200	5.855	5.814(99.3%)	5.648(96.5%)	39.166(99.9%)	5.842(99.8%)	5.803(99.3%)	5.640(96.5%)	49.59%
W_HPC15	39.938	5.964	5.917(99.2%)	5.724(96.0%)	39.904(99.9%)	5.952(99.8%)	5.907(99.3%)	5.716(96.0%)	49.54%
